# Non-participation in chlamydia screening in the Netherlands: determinants associated with young people’s intention to participate in chlamydia screening

**DOI:** 10.1186/1471-2458-13-1091

**Published:** 2013-11-23

**Authors:** Gill A ten Hoor, Robert AC Ruiter, Jan EAM van Bergen, Christian JPA Hoebe, Katrijn Houben, Gerjo Kok

**Affiliations:** 1 Department of Work & Social Psychology, Maastricht University, P.O. Box 616, 6200MD Maastricht, The Netherlands; 2 STI AIDS Netherlands, Keizersgracht 390, 1016GB Amsterdam, The Netherlands; 3 Department of General Practice, AMC-University of Amsterdam, P.O. Box 19268, 1000GG Amsterdam, The Netherlands; 4 Department of Sexual Health, Infectious Disease and Environmental Health, South Limburg Public Health Service, P.O. Box 2022, 6160HA Geleen, The Netherlands; 5 Department of Medical Microbiology, Research School CAPHRI, Maastricht University Medical Centre (MUMC+), P.O. Box 5800, 6202AZ Maastricht, The Netherlands; 6 Department of Clinical Psychological Science, Maastricht University, P.O. Box 616, 6200MD Maastricht, The Netherlands

**Keywords:** Chlamydia screening, Participation, Non-response, Determinants, Implicit associations

## Abstract

**Background:**

In the Netherlands, a national chlamydia screening program started in 2008, but the participation was low and the screening was not cost-effective. This study aimed to explore unconscious and conscious associations with chlamydia screening (16-29 year-olds). In addition, we examined whether information presented in chlamydia screening invitation letters had an effect on the evaluation of these determinants compared to a no-letter group.

**Methods:**

An Internet survey was conducted that included self-report measures of attitude, susceptibility, severity, unrealistic optimism, subjective, moral, and descriptive norm, perceived behavioral control, outcome expectations, barriers, intention, and a response time measure to assess unconscious associations of chlamydia screening with annoyance, threat and reassurance.

**Results:**

On the unconscious level, participants (N = 713) who received no information letter associated testing for chlamydia with annoyance and threat, but also with reassurance (all *p’s <* .001). On the self-report measures, participants showed a low intention towards chlamydia screening (*M* = 1.42, range 1–5). Subjective norm, moral norm, perceived susceptibility and attitude were the most important predictors of the intention to screen (R^2^ = .56). Participants who rated their susceptibility as high also reported more risky behaviors (*p* < .001).

In the groups that received a letter (N = 735), a weaker unconscious association of chlamydia screening with annoyance was found compared with the no-letter group (*p* < .001), but no differences were found in reassurance or threat. Furthermore, the letters caused a higher intention (*p* < .001), but intention remained low (*M =* 1.74). On a conscious level, giving information caused a more positive attitude, higher susceptibility, a higher subjective and moral norm, and more positive outcome expectations (all *p’s* < .001).

**Conclusion:**

Subjective norm, moral norm, susceptibility, and attitude towards chlamydia might be crucial targets to increase chlamydia screening behavior among sexually active young people. This study shows that informational invitation letters increase the intention and the intention-predicting variables. More evidence is needed on whether screening behavior can be increased by the use of an alternative information letter adapted to the specific unconscious and conscious determinants revealed in this study, or that we need other, more interactive behavior change methods.

## Background

Chlamydia screening has been promoted as a means to control chlamydia, the worldwide’s most prevalent bacterial sexually transmitted infection causing most cases of infection-related female infertility worldwide [1]. In April 2008, a pilot annual chlamydia screening program started in three Dutch regions. Sexually active young adults between 16 and 29 years were invited by the Public Health Services (PHS’s) to participate. Via a letter to their home address (based on municipal registries), they were asked to visit a website (http://www.chlamydiatest.nl) to anonymously request a test package. They could then do a chlamydia test at home, send it to a laboratory and within two weeks, they were able to privately review their test results online and could be treated by general practitioners or PHS’s. Despite the relative simple and costless procedure, the participation rate in this screening program was lower than expected and lowered during each round (16.1% in the first round; 10.8% in round 2, 9.5% in round 3). National implementation of the program was therefore evaluated as not cost-effective [2,3].

Chlamydia is the most reported sexually transmitted disease in the Netherlands [4], but insights into why people are not participating in chlamydia screening programs are limited. In a quantitative study, Greenland and colleagues [5] found that sexually active non-participants in the Dutch Ct-screening considered the risk of infection as low, and reported no time or interest as reasons for non-participation (barriers). In a qualitative Dutch study [6] examining why people in the Dutch Ct-screening did not request a test package, or did not resend their test package to the laboratory, ‘not having time’ (barrier), ‘being afraid of doing it wrong’ (threat), ‘the unpleasant procedure’ (annoyance), or ‘afraid for the consequences’ (threat) were the most important reasons. In both studies, the response from non-participants was low and the mentioned reasons did not sufficiently clarify the low response rate in the Dutch chlamydia screening program.

Various earlier international studies report that people feel invulnerable to infection, and compare themselves favorably with people who get infected (unrealistic optimism) [7]. In the New Orleans school-based screening for chlamydia and Gonorrhea [8], 3336 students who provided urine specimens for testing were asked about their chances of being infected with a sexually transmitted disease. Of 1183 students who were categorized as perceiving themselves at high risk, 12.8% tested positive for chlamydia compared with 11.4% among 2153 students who were categorized as not perceiving themselves at high risk (no significant difference).

Other studies identified lack of knowledge and guidance as possible barriers for participation [9] next to anticipated fear of the test result and negative reactions from the partner and others [10,11], with women expressing more anxiety for future fertility and men reporting more avoidance or blame to their female sex partner [12]. In addition, pilot studies show that populations with a higher risk have a higher intention to test when test kits are easily available [2,4,13].

Contemporary dual process theories suggest that behavior is determined by the interplay of two qualitatively different systems: a fast, associative, implicit, *impulsive* system, which includes automatic appraisal of stimuli in terms of their affective and motivational significance, and a slower, rule-based, explicit, *reflective* system, which includes controlled processes related to intentions, conscious deliberations, emotion regulation, and expected outcomes [14]. The impulsive and reflective system can trigger simultaneous, conflicting signals, but ultimately, behavior is determined by the relative strength of impulsive and reflective reactions in the sense that stronger reactions gain advantage over weaker ones [14].

**Figure 1:**
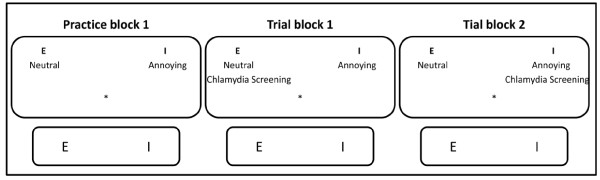
**The Single Category Implicit Associations’ Test (SC-IAT).** Single Category Implicit Association tests (SC-IATs) are computerized sorting tasks in which, based on reaction times, impulsive preferences are measured. Very simplified in the Figure 1 (three times a computer screen with a keyboard – the ‘E’ and ‘I’-button emphasized), the procedure of a SC-IAT is displayed. The SC-IAT consists of a practice block, and two trial blocks. In a first practice block, participants learn to respond to attribute words (appearing in the center of the computer screen) which have to be categorized in two categories. In this example the attribute categories are ‘neutral’ and ‘annoying’, and are positioned on the left and right side at the top of a computer screen. The attribute words (see Table 1) that appear one by one in the center of the computer screen have to be classified to the attribute categories with either the left (E) or right (I) response key. Participants are to assign the word as fast and accurate as possible to the correct category by pressing the appropriate response key. The assignment of the left key (E) and right key (I) are balanced across participants. The attribute words can be neutral, negative or positive; in this study: annoying, reassuring, and threatening. In the two following blocks, people continue categorizing attribute words, but now the label category ‘Chlamydia screening’ is added to either one of the categories. In block 2, ‘Chlamydia screening’ is combined with ‘neutral’, while in block 3 ‘Chlamydia screening’ is combined with ‘annoying’ and vice versa. If, for example, there is a significant higher mean sorting reaction time when ‘Chlamydia Screening’ is combined with ‘Annoying’ compared to when it is combined with ‘Neutral’, respondents have an impulsive negative reactance towards Chlamydia screening; they associate Chlamydia screening more with the attribute annoying than with a neutral attribute.

From this perspective, it follows that to understand the reasons for (non-)participation in chlamydia screening, it is necessary to measure both impulsive (or implicit) and reflective (or reasoned) reactions to chlamydia testing. While reasoned reactions can be relatively easy measured by self-reports, implicit reactions need to be measured indirectly. Indirect measures, such as the Single Category Implicit Association Test (SC-IAT) do not rely on self-report, but infer reactions from performance on reaction time tasks (see Figure 1) [15]. The SC-IAT is a computerized sorting task that infers implicit associations from the simultaneous classification of one target category, for example words related to chlamydia testing, and two affective attribute categories, for example ‘threatening’ versus ‘neutral’. Words representing all three categories are presented one at a time and participants must categorize them as quickly as possible. In one of the two key conditions, words related to chlamydia testing or representing ‘threatening’ are categorized with one response (a key press), and words representing ‘neutral’ are categorized with an alternative response. In the other condition, chlamydia words and ‘neutral’ words are categorized with one response, and threatening words with the other. The difference in average reaction time between these two conditions reflects the strength of association between the target and the attribute. For example, if people react faster to the categorization of chlamydia with ‘threatening’ than to the categorization of chlamydia with ‘neutral’, we infer an implicit association of chlamydia with threatening over the neutral.

In this cross-sectional study, the first aim is to gain more insights in determinants of non-screening behavior, by examining both implicit associations [15,16] and reasoned reactions towards chlamydia screening. Determinants in this study are defined as factors that have been found to be associated with intention and behavior and can be hypothesized to influence the behavior. Earlier qualitative studies showed that giving verbal or written information about chlamydia infection, treatment, and possible effects caused both a reassured feeling but also anxiety and annoyance [7]. The second aim is therefore to unravel the influences of giving information by a letter, on the implicit and reasoned determinants and intentions to participate in chlamydia screening.

## Methods

Following recent pleas for full disclosure of research materials [17,18] data, analyses and output, the informed consents, the questionnaires (in Portable Document Format, .pdf), and the letters (in Dutch, in PDF), are combined in a .rar archive as Additional file 1 and in a scientific repository at http://sciencerep.org/10/.

### Participants

From a random sample, 3716 participants (2732 female) between the ages of 16 to 29 years were invited through Flycatcher, a representative online panel (http://www.flycatcher.eu/). The Flycatcher panel consists out of 16.000 individuals above 12 years of age, and is representative for the Dutch population (ISO 26362 Dutch quality label, certifying that the panel can be used for social-scientific research). In total, 1822 subjects completed the study (49% response rate). For analyses, participants who received a letter to test for chlamydia in the past (N = 273), or participants who actually have tested for chlamydia in the six months before this study (N = 165) were excluded.

### Procedure

To find out what the determinants of non-screening behavior are, and to examine whether the original informational invitation letter had an impact on impulsive associations and reasoned reactions, participants were randomly allocated to three different groups. The first aim was studied in the no-letter group. For the second aim, we compared the influence of the original PHS invitation letter with the no-letter group, but we also developed an alternative letter. In this alternative letter, the informational content was similar to the original PHS letter but the content was simplified and adapted to decrease possible negative associations and promote a more positive attitude towards chlamydia (see *The invitation letters*). Participants in the first group (25%) were asked to start with reading the newly developed letter. In the second group (25%), subjects read the original invitation letter of the PHS’s. The third (control) group (50% of all participants) did not receive any information at the start of the study. Through the letters, young adults were invited to participate in the annual chlamydia screening, and information was given about the chlamydia screening including general information, the purpose and procedures (the participants in this study were told that they were not in fact invited to test for chlamydia but to just evaluate the letter).

**Figure 2:**
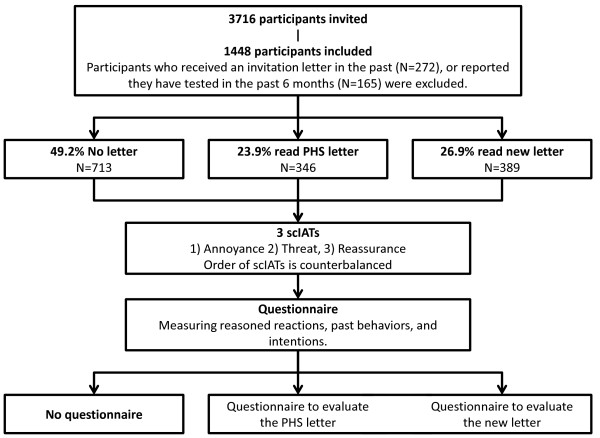
Flowchart illustrating the procedure of the study and the number of participants.

After giving informed consent by clicking on the appropriate button (see also Additional file 1 for the original and translated informed consent), participants were randomized. Subsequently, the participants in the letter-groups read the invitation letter, after which the implicit associations were measured; the no-letter group started after randomization immediately with the implicit association measures. Then, all participants received a self-report questionnaire in which reasoned reactions, including intentions, and past behaviors were measured. Reasoned reactions were measured last because their measurement may influence implicit associations. Finally, people in the two letter-groups received another short questionnaire to evaluate the letter they read, after which all participants were debriefed (see Figure 2). At the beginning of the study, participants were informed about the procedures but not about the content of the study, as associations and answers might be influenced. This study was approved by the Research Ethics Board of the Faculty of Psychology and Neuroscience, Maastricht University.

#### The invitation letters

In the two letter groups, participants read either the PHS letter, or the newly developed letter before the measure of implicit associations. Through the PHS letter young people aged 16–29 were invited to participate in the annual chlamydia screening. It is explained that chlamydia is a sexually transmitted disease with a high prevalence in young people, that it may have severe consequences without treatment, but also that it is easy treatable, and that people often have chlamydia without noticing it. Furthermore an explanation of how to get tested was given, a personal login code, information about the procedure, and that all information was treated confidentially.

The new letter was similar in lay out, but the content was adapted based on appropriate behavior change theories [19-23]. To keep this letter short, readers were referred to the website http://www.chlamydiatest.nl for detailed procedural aspects. Logos were identical but fonts were slightly larger to improve readability (see Additional file 1, or the scientific repository at http://sciencerep.org/10/ for the Dutch letters used in this study).

### Measures

#### Implicit measures

Implicit associations were measured using three SC-IAT’s [15], see Figure 1.

In an earlier study by the PHS Amsterdam [6], it was found that the most important reason to request a chlamydia test package was the perceived feeling of reassurance. Reasons for not requesting or resending the test package were the ‘feeling of doing it wrong’, the consequences of a negative outcome (threatening), and the ‘unpleasant’ or ‘unclear’ procedure (annoying). Based on these outcomes, the three SC-IAT’s had the attribute category ‘Neutral’ and either 1) Reassuring, 2) Threatening, or 3) Annoying. The used ‘sorting-words’ for the label category and the different attributes are displayed in Table 1.

**Table 1:** Used words for the label category “Chlamydia screening” and the attribute categories “Neutral”, “Reassuring”, “Annoying”, and “Threatening”

	**Chlamydia screening**	**Neutral**	**Reassuring**	**Annoying**	**Threatening**
**Stimuli**	Chlamydia test	Normal	Safe	Irritating	Unsafe
	Chlamydia screening	Standard	Secure	Bothersome	Insecure
	Test package	Common	Protected	Frustrating	Scared
	Screening	Usual	Sure	Difficult	Afraid

#### Explicit measures

The questionnaire consisted of four parts. In the first part, gender, age, educational status, whether participants had been tested for chlamydia in the past six months, and whether they ever received an invitation for chlamydia screening were recorded with appropriate self-report items.

The second part measured 10 social cognitive variables (including intention) with 27 items in total (See Table 2). The variables for the questionnaire were selected from theories on risk perception (e.g. [22]) and general social cognitive theories (e.g. [20,21]) and are hypothesized to predict human behavior. All items were rated on a 5-point Likert scale ranging from (1) *completely disagree* to (5) *completely agree*, unless otherwise stated. Scores on items that measured the same construct were averaged into one scale where internal consistency was sufficient (α > .60). Scores were recoded such that a higher score reflected a higher value of the variable.

**Table 2:** Questionnaire with social cognitive determinants

**Determinant**	**Number of items**	***r *** **or Cronbach's alpha**	**Example question**	**All questions were rated ‘completely agree – completely disagree’, except for:**
*Intention*	2	.81	*I will do a chlamydia test in the coming three months;*	Question was rated ’not very probable - very probable’
*Attitude*	5	.73	*When I take a chlamydia test, for me that would be..*	Questions were rated ’very unpleasant - very pleasant’, very annoying - very enjoyable’, an extreme bad idea - an extreme good idea’, very unimportant - very important’, and ’very threatening - very safe’.
*Susceptibility*	4	.68	*It is possible that I’ve had sex with someone with chlamydia*	
*Severity (1–8)*	1	-	Eight different health issues had to be ranked from 1 to 8 based on how severe it would be to get the health issue within 12 months. The eight health issues were ’hearing impairment’, “Pfeiffer disease”, “chlamydia”, “Repetitive Strain Injury (RSI)”, “Lyme disease”, “Caries”, “Heart attack”, and “HIV/AIDS”.	
*Unrealistic optimism*	2	.65	*The chance that I will get chlamydia compared to someone of the same gender and age is…*	Question was rated ’much larger - much smaller’
*Subjective norm*	3	.82	*My family thinks that I should get a chlamydia test in the coming three months*	
*Descriptive norm*	3	.88	*My friends takes a chlamydia test*	
*Moral norm*	2	.70	*It would be wrong if I don’t take a chlamydia test*	
*Perceived behavioral control*	3	.87	*If I want to, I’m sure that I can do a chlamydia test in the coming three month.*	
*Outcome expectations*	2	.77	*If I would take a chlamydia test, it would give certainty about my health*	Question was rated ‘very unlikely - very likely’
*Barriers*	3	-	Three potential barriers (mentioned in earlier studies [5,6] for non participation were questioned: the barrier of having no time, no internet access, or the barrier of not willing to test (‘don’t feel like testing’).	

n the third part of the questionnaire, past behaviors were asked as an estimation of the true risk: 1) the number of sexual partners ever (0, 1, 2, 3–5, 6–10, >10), and 2) whether sexual contact was always with a condom (always, most of the time, sometimes with a condom, most of the time without a condom, never with a condom).

The last part of the questionnaire (only for the letter groups) consisted of 11 questions regarding the level of understanding and completeness of the recently read invitation letter and attitudes towards requesting the package, chlamydia testing, sending back the test package, and requesting the results.

#### Analyses

IBM SPSS statistics 20 was used to analyze the data. For the implicit measures, d-600 scores were calculated using the d-600 algorithm [24]. The d-600 score calculates the difference in reaction times, corrected for incorrect responses where higher scores indicate stronger associations of chlamydia screening with either ‘annoying’, ‘reassuring’ or ‘threatening’ compared to associations with ‘neutral’. One sample t-tests were used to see whether the d-600 scores significantly differed from 0.

Descriptive analyses - means (M) and standard deviations (SD) - were conducted to provide an overall picture of the sample. Differences between the no-letter, PHS letter and new letter group were calculated using between-subject factorial ANOVA’s with the factor ‘group’ and the d-600 scores and explicit measures as dependent variable. Comparisons were two-tailed and considered as statistically significant at *p* < .05.

To determine the relationship between intention and the selected reasoned reactions, correlations were calculated using Pearson’s correlation coefficient. To determine significant determinants of intention to be screened for chlamydia, backward, stepwise multivariable regression models were fitted to assess the explained variance in the intention to participate in the chlamydia screening campaign as a result of including significant unique correlations in the prediction model.

## Results

From the 1822 participants who completed the study, data from 1448 participants were used for further analyses as 374 participants already had either received an invitation letter in the past (n = 273) and/or were tested for chlamydia in the past six months (n = 165). The three groups did not differ in gender, age or education level (p’s > .18; see Table 3 for frequencies).

**Table 3:** Descriptives of study population

	**Total**	**New letter**	**PHS letter**	**No letter**
N	1448	389	346	713
Age (SD)	24.1 (3.5)	24.0 (3.6)	23.9 (3.4)	24.3 (3.4)
Gender (male:female)	370:1078	103:286	88:258	179:534
education level				
none or primary school	58	22	9	27
Pre-vocational secondary education (VMBO)	81	21	27	33
Secondary vocational education (MBO)	197	51	39	107
Senior general secondary education (HAVO) or Pre-university education (VWO)	382	105	96	181
Higher professional education (HBO)	264	76	70	118
University education (WO)	466	114	105	247

No significant difference between groups. All p’s > .17.

### Determinants of non-screening behavior (No-letter group)

Two questions were not used for further analyses because of the skewness. The first question “do you think you can test for chlamydia when you don’t have access to the internet?” (*M =* 3.89) was irrelevant as nowadays almost 100% of 16–29 year olds in the Netherlands have access to the internet [25]. The second question that is not used for further analyses is “do you think you can test for chlamydia when you don’t have time” (*M =* 1.63). This question was multi interpretable and not exactly measuring what we intended (i.e., the willingness to participate when people were busy).

Table 4 presents the mean of the determinants, correlation and regression coefficients with intention to screen. One sample t-tests in the no-letter group show that the d-600 scores for reassuring, threatening, and annoying significantly differed from 0 indicating that participants associated chlamydia screening significantly more with reassurance, threat and annoyance than with neutral (*t =* 20.53, *t =* 11.89, and *t =*20.37 respectively, all p < .001).

**Table 4:** Means, correlation coefficients with intention to screen and R-squares for the explicit and implicit measures

	**No letter**		**Letter (PHS + New)**		**No letter vs. letter**
	**(N = 713)**		**(N = 735)**		
	***M (SD)***	***r***	***M (SD)***	***r***	***p***
***Implicit***					
**D-600 reassured**	0.25 (.32)	.05	0.29 (.32)	-.04	.05
**D-600 threatening**	0.16 (.35)	.01	0.13 (.32)	-.05	.05
**D-600 annoying**	0.25 (.32)	-.03	0.17 (.33)	-.00	<.001
***Explicit***					
**Intention to screen**	1.42 (.76)	1.00*	1.74 (.94)	1.00*	<.001
Attitude**	2.65 (.65)	.35*	2.99 (.64)	.40*	<.001
Susceptibility**	1.58 (.69)	.53*	1.75 (.77)	.46*	<.001
**Severity of chlamydia (1–8)**	5.08 (1.39)	-.07	5.26 (1.38)	-.07	.01
**Unrealistic optimism**	3.15 (.81)	-.42*	3.08 (.82)	-.36*	.08
Subjective norm**	1.18 (.53)	.65*	1.30 (.66)	.50*	<.001
**Descriptive norm**	1.95 (1.09)	.44*	2.05 (1.08)	.36*	.09
Moral norm**	2.07 (1.08)	.53*	2.36 (1.11)	.59*	<.001
**Perceived behavioral control**	4.22 (.93)	.01	4.25 (.86)	.00	.66
**Outcome expectations**	3.22 (1.21)	.26*	3.44 (.1.14)	.35*	<.001
**Barrier – don’t feel like taking it**	3.28 (1.39)	-.03	3.19 (.1.36)	-.00	.19
**R**^**2**^		.56		.49	

*correlation with intention: p < .001 (Bonferroni correction for multiple comparisons).

**significant predictors of intention in the multivariate linear model.

M = mean; (SD) = Standard Deviation. The D-600 scores are on a continuum, where a higher score indicates a more positive association with the variable. All explicit measures, except Severity, are from 1–5, where 5 indicates a higher score on the variable.

The explicit measures showed a low score for intention to participate in chlamydia screening. This intention was positively correlated with the subjective norm, the moral norm, susceptibility, the descriptive norm, one’s attitude, and outcome expectations, and negatively with unrealistic optimism, but not with implicit associations (Table 4). Attitude (*β* = .09), susceptibility (*β* = .20), subjective norm (*β* = .61), and moral norm (*β* = .19) were significant (positive) predictors, and predicted 55% of the intention to participate in chlamydia screening. The severity of chlamydia, perceived behavioral control, and the barrier ‘don’t feel like taking a chlamydia test’ were not correlated to one’s intention.

### Influence of information on determinants of non-screening behavior

#### Evaluation of the letters

Table 5 presents the means of the evaluation of the letters. The two letters were evaluated similarly: no differences were found in clearness, understandability, intention to visit the website, importance to visit the website and attitude towards visiting the website, requesting a test package, doing a chlamydia test, sending back the test package or requesting the results of the chlamydia test. Only the completeness of the original PHS letter was rated slightly higher than in the new letter *t* (733) = 2.19, *p* = .03.

**Table 5:** Evaluation of the letters (range: (1) not at all – (5) completely)

	**New letter (N = 389)**	**PHS letter (n = 346)**	**p**
	***M (SD)***	***M (SD)***	
**Clearness**	4.43 (.74)	4.39 (.83)	.41
**Completeness**	4.10 (.84)	4.22 (.73)	.03
**Understandability**	4.43 (.68)	4.42 (.70)	.84
**Intention to visit the website**	2.22 (1.33)	2.26 (1.28)	.73
**Importance of visiting the website**	2.51 (1.23)	2.57 (1.23)	.57
**Attitude towards chlamydia testing**	3.29 (.79)	3.23 (.75)	.30
**Attitude towards sending back a chlamydia test.**	3.70 (.96)	3.78 (.97)	.25
**Attitude towards requesting the results of the chlamydia test**	3.74 (.86)	3.79 (.80)	.47

There were no significant differences between the PHS letter and the newly developed letter groups on both implicit and explicit measures. Therefore, the two letter groups were combined for further analyses (see Table 4). Differences were found between the no-letter group and the combined letter group. Receiving a letter resulted in a higher score on the implicit measure annoyance (*t* (1428) = 4.69, *p* < .001), a higher intention (*t* (1444) = -7.23, *p* < .001), a higher attitude (*t* (1446) = -10.02, *p* < .001), more susceptibility (*t* (1439) = 4.41, *p* < .001), a higher subjective norm (*t* (1228) = -3.64, *p* < .001), a more positive moral norm (*t* (1446) = -5.09, *p* < .001), and more positive outcome expectations (*t* (1434) = -5.54, *p* < .001).

In the letter groups the intention to request a test package was (positively) predicted by the same determinants as in the no-letter group (i.e. attitude (*β* = .16), susceptibility (*β* = .19), subjective norm (*β* = .28), and moral norm (*β* = .35)) by 47%.

### True risk versus perceived risk

Based on the self-reported number of sex partners ever and condom use, a risk score was calculated (see Table 6). People with one sex partner, *and* who reported to always use condoms during intercourse, were considered the low risk group (N = 143). People with two or more sex partners, *and* who reported to not always use condoms (N = 91) were considered being the high risk group.

**Table 6:** **Self-monitored risk behavior (*N*** **= 1448)**

	
***Partners ever***	***N***
0 partners	376
1 partner	953
2 partners	73
3 - 5 partners	33
6 - 10 partners	8
More than 10 partners	5
***Condom use***	***N***
Always	171
Most of the time	111
Sometimes	70
Most of the time without	142
Never	578
(No partner)	376

Analyses of variance showed that the low-risk group differed from the high-risk group by a lower intention (*M* low risk = 1.59, *SD* = .80; *M* high risk = 2.49, *SD* = 1.30), *F* (1,234) = 42.43*, p* < .001*, η*_*p*_^*2*^ = 0.16 and lower perceived susceptibility (*M* low risk = 1.67, *SD* = .71; *M* high risk = 2.58, *SD* = .94), *F* (1,234) *=* 68.64, *p* < .001, *η*_*p*_^*2*^ = 0.23. In other words, participants with high-risk behavior also have a higher perceived susceptibility and a higher intention to test. There was no interaction effect with receiving a letter or not for intention, *F* (1,234) = 1.50, *p* = .22 *η*_*p*_^2^ = 0.006, nor for perceived susceptibility, *F* (1,234) = 0.64, *p* = .43 *η*_*p*_^2^ = 0.003.

## Discussion

In this study, we examined determinants of chlamydia screening behavior by examining both implicit associations and reasoned reactions towards chlamydia screening in a population not recently tested for chlamydia. Furthermore we examined the influences of giving information by two different letters, on the implicit and reasoned determinants and intention to participate in chlamydia screening.

Although participants state that they are able to test for chlamydia, participants do not have the intention to test. Possible (logic) explanations of why people are not testing may be first, the low perceived susceptibility [26], and second, the annoyance related to testing: asking a young person to log on to a website, wait for a test kit, collect a specimen and mail it back does not fit well with young people’s sense of immediacy or desire for instant gratification.

We found support for the first, but not for the second explanation. Results show that people have ambivalent feelings towards chlamydia screening - they associate chlamydia screening with annoying and threatening, but also with a reassured feeling. As expected, these associations were not correlated with one’s intention, because implicit associations are supposed to influence behavior without mediation by intentions. Follow-up research with actual behavior may provide better answers about the effects of implicit associations next to reasoned reactions on screening behavior. We found that the most important determinants of the low intention are low susceptibility, a low moral norm, and no supportive social norms.

For the Dutch 16–29 year olds, the not recently tested population, this study provides answers about the most important target determinants for improving chlamydia screening in the future. However, causality cannot be shown in cross-sectional studies. Moreover, it is not easy to make statements about the effects on actual screening behavior. A limitation is that this study focused on the behavioral intention and not the behavior itself. Therefore, more research is needed on whether the ambivalence in implicit reactions to chlamydia screening can be confirmed with a behavioral outcome, and how the ambivalence can be steered in a more positive direction. This study shows that one way to significantly increase the intention and the intention-predicting variables to screen is giving informational invitation letters. Moreover, assuming that low susceptibility is the cause of a low intention, more research is needed on whether there is a discrepancy between perceived susceptibility and real susceptibility and what the possible reasons for this discrepancy are [27].

Furthermore, more evidence is needed on whether screening behavior can be increased by the use of an alternative information letter adapted to the specific implicit and reasoned determinants revealed in this study, or that we need other, more interactive behavior change methods e.g. the use of Internet independent of geographic area [28], financial incentives [29], a focus on self-identity [30], and/or the use of social media in combination with role models (people who disclose that they have tested) to overcome low susceptibility and lack of supportive social norms [31].

## Conclusion

In conclusion, this study adds to the existing knowledge about determinants of chlamydia testing intention and behavior as summarized in the introduction. Participants should become more aware of their susceptibility. An informational letter used in the original PHS campaign is effective in increasing one’s intention to participate in chlamydia screening, however obviously not enough as the intentions are still low.

## Abbreviations

PHS: Public Health Service; SC-IAT: Single Category Impliciat Association Test.

## Competing interests

The authors declare that they have no competing interests.

## Authors’ contributions

RR, GK, JB and CH conceived the study; GH, RR, GK and KH participated in the design of the study; GH carried out the study and performed the analyses; GH, RR, GK and KH contributed to the interpretation of the data; GH and GK drafted the manuscript; and all authors read the manuscript, provided comments and approved of the final manuscript.

## Pre-publication history

The pre-publication history for this paper can be accessed here:

http://www.biomedcentral.com/1471-2458/13/1091/prepub

## Supplementary Material

Additional file 1 Invitation letters, questionnaires, databases and syntax for the study ‘Non-Participation in Chlamydia Screening in the Netherlands: Determinants associated with young people's intention to participate in chlamydia screening. Click here for file
